# Comparison of the Percentage of Voids in the Canal Filling of a Calcium Silicate-Based Sealer and Gutta Percha Cones Using Two Obturation Techniques

**DOI:** 10.3390/ma10101170

**Published:** 2017-10-12

**Authors:** Sohee Kim, Sunil Kim, Jeong-Won Park, Il-Young Jung, Su-Jung Shin

**Affiliations:** 1 Department of Conservative Dentistry, Yonsei University College of Dentistry, Gangnam Severance Hospital, 211 Eonjuro, Gangnam-gu, Seoul 135-720, Korea; sohee928@yuhs.ac; 2 Department of Conservative Dentistry and Oral Science Research Center, Yonsei University College of Dentistry, 50 Yonsei-ro, Shinchon-dong, Seodaemun-gu, Seoul 120-752, Korea; seone1@yuhs.ac (S.K.); juen@yuhs.ac (I.-Y.J.); 3 Department of Conservative Dentistry and Oral Science Research Center, Yonsei University College of Dentistry, Gangnam Severance Hospital, 211 Eonjuro, Gangnam-gu, Seoul 135-720, Korea; pjw@yuhs.ac

**Keywords:** canal filling, endoseal MTA sealer, microcomputed tomography, sealing, single cone technique

## Abstract

This study evaluated the root-filling quality of a calcium silicate-based sealer and gutta percha (GP) cones by measuring the percentage of voids. Twenty artificial molar teeth were divided into two groups: one obturated using the single-cone (SC) technique, and the other using the continuous wave (CW) technique. Obturation was performed with GP cones and Endoseal MTA (mineral trioxide aggregate, Maruchi, Wonju, Korea). Obturated teeth were scanned using microcomputed tomography, and the percentage of void volume was calculated in the apical and coronal areas. A linear mixed model was used to determine the differences between the two techniques (*p* < 0.05). The percentage of voids between the filling materials and root canal walls was not significantly different between the two obturation methods (*p* > 0.05), except for the CW group, which demonstrated a significantly higher void volume in the coronal area of the distal canal (*p* < 0.05). The percentage of voids *inside* the filling material was significantly higher in the CW groups for all of the comparisons (*p* < 0.05), except in the apical area of the distal canal (*p* > 0.05). The voids between the filling material and canal wall in the apical area were not significantly different between the two techniques.

## 1. Introduction

Root canal obturation is a procedure in which the root canal space is filled with canal-filling materials at the final stage of root canal treatment, after cleaning and shaping. The objective of root canal obturation is to prevent or treat periapical disease by preventing recontamination by bacteria that may have remained in the dentinal tubules or that exist in the oral cavity [1]. Previous studies have demonstrated that the quality of root canal obturation affects periapical healing and treatment success [2,3]. Root canals often have a complex anatomy, including lateral canals and an isthmus [4]. Isthmus communication in the mandibular first molar exists in 54.8% of cases [5]. Although a few studies [6,7] have used human molar teeth to evaluate the amount of voids in root canal fillings, the variation was large because of complex canal shapes [4,5]. For these reasons, artificial resin teeth can result in more reliable findings when measuring the void percentage.

To achieve high-quality obturation, it is important to select a sealer that can provide good adhesion to the root canal walls and penetrate the dentinal tubules [8]. Calcium silicate-based sealers composed of calcium silicate and/or calcium phosphate have recently been developed, and their physical and chemical properties and biocompatibility have been intensively studied [9,10,11,12]. Several studies have demonstrated that calcium silicate-based sealers are biocompatible, less toxic [9,10], non-shrinking [11], and chemically stable within a biological environment [11]. They also have the advantage of self-sealing by forming hydroxyapatite with the canal wall during the setting process, which enhances sealability [9,12], and ultimately creates adhesion between the root dentin and the filling material. Endoseal MTA sealer (Maruchi, Wonju, Korea) is an injectable premixed bioceramic endodontic sealer that was recently introduced to the market [13]. This product consists of calcium silicates, calcium aluminates, calcium aluminoferrite, calcium sulfates, a radiopacifier, and a thickening agent.

The traditional single-cone (SC) obturation technique has been used to fill the canal with a sealer and one gutta percha cone. However, it is known that the SC technique without apical pressure is inferior to lateral or vertical compaction techniques [14]. Recently, one manufacturer of calcium silicate-based sealers recommends using them with the SC technique [15]. The SC technique is less likely to exert excess stress on the tooth and, therefore, leads to fewer apical cracks [16]. In addition, the SC method is easier to implement; consequently, it is less sensitive to variations in technique or operator ability. In a previous study, it was reported that the SC technique demonstrated a higher percentage of voids than the continuous wave (CW) technique [7]. Moreover, a sealer may not penetrate finer gaps such as the isthmus or dentinal tubules. However, newly introduced calcium silicate-based sealer has different viscosity, flowability and sealability. Therefore, further studies are needed.

With this background, the aim of this study was to assess the quality of root filling using a calcium silicate-based sealer and gutta percha (GP) cones by calculating the percentage of voids when using the SC or CW filling techniques on artificial molars with microcomputed tomography (micro-CT). The null hypothesis was that the SC technique using a calcium silicate-based sealer would demonstrate a root canal filling quality similar to the conventional CW technique.

## 2. Results

**Figure 1:**
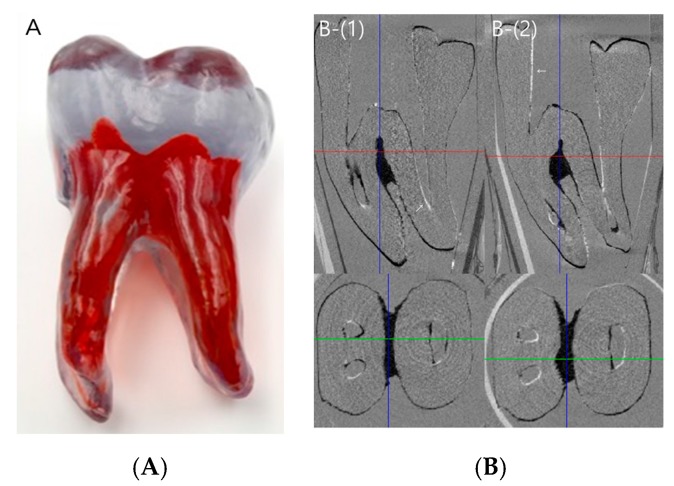
Artificial teeth. (**A**) Artificial molar teeth used in this experiment; (**B**) Superimposition images of the prepared canal spaces before canal obturation. The white line in the canal space (arrow) indicates a difference between samples. (1) The overlapping image of samples 1 and 2. (2) The overlapping image of samples 1 and 3.

**Figure 2:**
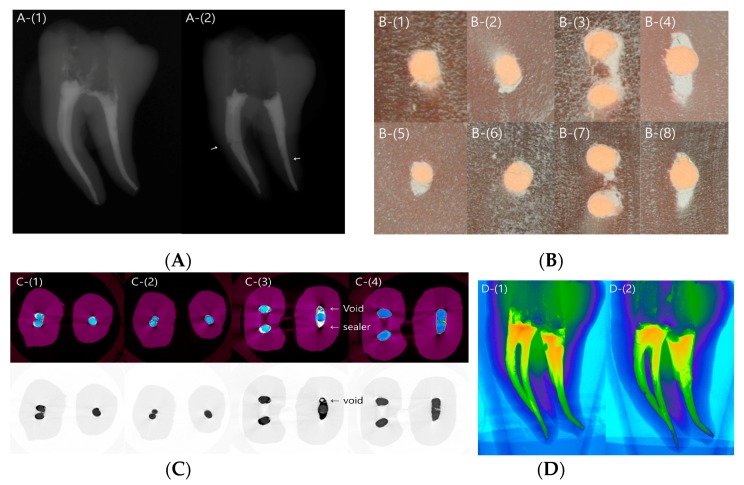
Microscopic and microcomputed tomography (micro-CT) images of the representative specimen. (**A**) Radiographic images after canal obturation using the single-cone (SC) (left panel) and continuous wave (CW) (right panel) techniques. Void formation is visible (arrow). (**B**) Microscope images in the sectioned area in (1) the apical area of the mesial canal in the SC group, (2) the apical area of the distal canal in the SC group, (3) the coronal area of the mesial canal in the SC group, (4) the coronal area of the distal canal in the SC group, (5) the apical area of the mesial canal in the CW group, (6) the apical area of distal canal in the CW group, (7) the coronal area of the mesial canal in the CW group, and (8) the coronal area of the distal canal in the CW group. (**C**) Representative micro-CT tomography images. The upper row is color-coded to distinguish the sealer from the gutta percha (GP). The sealer, void, and gutta percha are marked by an arrow. The image shows (1) the apical area in the single-cone (SC) group, (2) the apical area in the continuous wave (CW) group, (3) the coronal area in the SC group, and (4) the coronal area in the CW group. (**D**) Three-dimensional reconstructed images. In the mesial canal, the SC and CW groups are generally packed well. In the distal canal, voids are visible in the coronal area in the SC group (1), and some loosely packed areas are visible in the CW group (2).

After canal preparation, the artificial tooth specimens were scanned (*n* = 3) in order to evaluate the shape of the prepared canal space and discrepancies among specimens (Figure 1A). Radiographs obtained after obturation revealed no obvious defects in the SC group, whereas voids were detected in some samples in the CW group (Figure 1B). The microscopic images at 20× magnification revealed that the apical and coronal sections in the SC and CW groups were well packed with gutta percha and sealer (Figure 2A,B).

Micro-CT revealed that the SC and CW groups were both well packed, without voids in the apical area. The coronal area in the SC group showed the gutta percha cone in the middle, with the surrounding sealer sealing the rest of the area. In the CW group, the gutta percha occupied most of the root canal space (Figure 2C,D).

The percentage of voids between the filling materials and root canal walls (%V_out_) demonstrated no statistically significant difference between the two obturation technique groups for all comparisons (*p* > 0.05), except in the coronal area of the distal canal (*p* < 0.05) (Table 1). The percentage of voids inside the filling material (%V_in_) was significantly higher in the CW group, for all comparisons (*p* < 0.05), except for the apical area of the distal canal (*p* > 0.05) (Table 2). Post hoc analysis revealed a significant difference between the two methods (Table 3).

In the images reconstructed three-dimensionally (3D), the mesial canal was generally well packed in the SC and CW groups. However, in the distal canal, voids were observed in the coronal area in the SC group, and some loosely packed areas were observed in the CW group (Figure 2C,D).

## 3. Discussion

We compared the void percentage, based on canal obturation technique, in artificial molar teeth. Most previous studies that have evaluated filling quality used premolars, or anterior teeth with a single canal and with few anatomical variations [8,12,17,18,19]. However, these samples may not have reflected the complex root canal anatomy encountered in clinical situations. A possible cause for endodontic failure after root canal filling is a complicated root canal shape, particularly the isthmus, which is common in molar teeth and cannot be properly treated. The artificial teeth used in the present study were custom-made, and had a Weine classification type II mesial canal, and one distal canal. To the best of our knowledge, this was the first study to use these artificial teeth in a micro-CT study.

Even if the same type of artificial teeth were used in this experiment, the prepared canal shapes may have been different, depending on the sample. Therefore, to evaluate whether the prepared canal shapes were consistent, three artificial teeth were selected from among the 20 artificial teeth after canal preparation. The images of three samples were superimposed using micro-CT. We found that the tooth-to-tooth variation was negligible.

The volume of voids has been used as a method of assessing root canal filling quality [20,21,22]. Non-obturated areas may allow bacteria to remain at a site and lead to treatment failure [23]. Therefore, proper canal obturation can prevent the penetration of microorganisms and toxins [24]. Unlike previous studies [6,7] that measured the amount of total voids in a canal filling, the evaluation of voids in this study used two categories. The first area was the voids between the canal filling material and root canal wall (i.e., V_out_). We also calculated the voids within the canal filling material (i.e., V_in_). In the V_out_, leakage can occur, and bacteria and/or bacterial by-products can penetrate. It was expected that the amount of V_out_ would directly affect the canal-filling quality, whereas V_in_ may have less effect on the actual leakage; however, their actual relationship with treatment outcome has yet to be determined.

In this study, there were more voids in the CW group than in the SC group, except in the apical area of the mesial canal. In the apical area of the mesial canal, there was no significant difference between the two groups. This may be because, unlike the SC technique, the CW technique includes a backfilling process after cutting the apical 5 mm area using the System B instrument, after inserting the master cone. In this process, if the tip of the backfilling device does not reach the cut-off point, voids may occur at the transition site. The tip reached the gutta percha segment and filled up to the 5 mm level, although the extruded gutta percha could not be packed tightly. Therefore, there was a possibility of voids forming at the transition site in the CW technique. The diameter of the distal canal was large in the coronal area; therefore, the gutta percha could not be filled in the large-diameter oval canal during the backfilling process. The backfilled gutta percha was condensed at the orifice level; thus, the pressure could not reach all the way down. We speculate these were reasons for the significantly higher percentage of voids in the CW group than in the SC group in the coronal portion. In our study, with regard to the V_out_ findings, more voids were formed in the CW group, for which the results were statistically significant only in the coronal area of the distal canal. Therefore, it is recommended that a large-diameter root canal be filled in two or three increments instead of backfilling the entire canal at once. However, there was no significant difference between the two groups in an apical area with a small canal diameter or small coronal part of the mesial canal.

In the present study, the void percentage was calculated by dividing the apical 1–5 mm of the root (i.e., the apical area) by the apical 5–9 mm (i.e., the coronal area). Previous studies have reported slightly different criteria for dividing the root area. Jho et al. [6] measured only the apical 5 mm area, because they considered this measurement to be more clinically relevant in root canal treatment success than measurements of the full canal length. Iglecias et al. [7] divided the root into three areas: apical, middle, and cervical. When confining the present results in the apical area to compare with other studies [7,23,25], our results were in agreement. Somma et al. [23] measured void distributions in root canal obturation in three different techniques in a straight canal in single-rooted permanent teeth using micro-CT, but found no significant difference between the Thermafil group, the SC group, and the System B group. In a similar study, Alshehri et al. [25] measured the volume of voids in the apical one-third of a curved canal in the mesial root of human mandibular first molars using micro-CT, and found similarities between the SC and CW groups. In addition, Iglecias et al. [7] measured the volume of voids in the mesial root of the human mandibular molar in the apical one-third, and found no significant difference between the SC and CW groups in the apical area.

This study had some limitations. The specimens were prepared by a single operator, who was a second-year resident and had used the CW technique for more than one year clinically. However, there was more deviation in the results from the CW group when we measured the number of voids in the coronal portion. Based on the results of the present study, the SC method appeared to be less sensitive to variations in operator technique or ability. In addition, it was difficult to accurately control the pressure that was applied during the gutta percha cone pumping for sealer application using the SC technique. Furthermore, there was no consistent guideline for this procedure. We believe the applied pressure may have affected the amount of sealer extrusion at the apical portion in the SC group. In fact, sealer extrusion often occurred in both experimental groups. Preventing sealer extrusion is important in clinical situations; therefore, the relationship between the pumping pressure and the amount of sealer extrusion should be further investigated. Another consideration in heating calcium silicate based sealers was the heat could alter the properties of the material. Camilleri demonstrated that heat application at 100 °C for 1 min altered the physical properties of an experimental tricalcium-based sealer [26].

In addition, there are some limitations to using plastic teeth in our experimental procedure. Calcium silicate cements have the advantage of self-sealing by the formation of hydroxyapatite with the canal wall during the setting process, which enhances sealability [1,2] and, ultimately, creates an adhesion between root dentin and the filling material. However, plastic teeth cannot reproduce this phenomenon. Additionally, plastic teeth do not have anatomical structures that are present in human teeth, such as dentinal tubules. Therefore, we were unable to examine the sealer penetration into dentinal tubules or hydroxyapatite formation between the sealer and root dentin.

## 4. Materials and Methods

### 4.1. Preparation of Plastic Tooth Samples

Twenty plastic artificial teeth (Dental Cadre, Santa Barbara, CA, USA) were used (Figure 1A). The plastic teeth were customized samples that reproduced the shape of a common human mandibular first molar. These teeth had access openings with a mesial root with a Weine classification type II canal [27], a curvature of 30 degrees [28], and a distal large oval canal with a curvature of 7° [28]. A K file (size #15; Dentsply Maillefer, Ballaigues, Switzerland) was inserted into the canal to achieve the working length (WL). The WL was 0.5 mm short of the apical tip point where the file came out and was visible at the apical foramen. The mesiolingual (ML) canal with a relatively smaller curvature was prepared to the WL, and the WL of the mesiobuccal (MB) canal was measured up to the point where the MB and ML canal met, which was 4 mm from the ML canal apex. The master apical file size was #40 for the MB and ML canals, and #50 for the distal canal. Medium size gutta percha cones (Dentsply Maillefer), customized to #40 for mesial and #50 for distal canals, were used, and the master cones were inserted into the WL or within 0.5 mm short of the WL. All of the canals were instrumented using the ProTaper Next NiTi System (Dentsply Maillefer). Between instrumentations, each canal was irrigated using distilled water and a 24-gauge needle (Korea Vaccine Co., Seoul, Korea). After the instrumentation was complete, all of the canals were dried using paper points (#25, Dentsply Maillefer). The teeth were then randomly assigned to one of two groups (*n* = 10 each) for canal obturation using one of the two techniques.

### 4.2. Preparation of Plastic Tooth Samples

#### Obturation of the Plastic Tooth Samples

***Single-cone technique group*:** Endoseal MTA sealer (Maruchi) was applied to the root canal using a 24-gauge needle tip provided by the manufacturer. The tip was slowly pulled back toward the orifice from the point where it became engaged. A gutta percha cone was inserted into the canal. The gutta percha cone was gently moved with an up-and-down-motion three times to allow the sealer to penetrate better into the fine structures. It was cut at the orifice level using System B (SybronEndo, Orange, CA, USA) and packed with Obtura S-Kondenser (Obtura Spartan, Earth City, MO, USA) in the coronal area.

***Continuous wave technique group*:** Using the same size gutta percha cones as the SC group, 3 mm of the end of the gutta percha cones were coated with Endoseal MTA sealer and inserted into the prepared root canals. Gutta percha was cut 5 mm from the apex using System B (SybronEndo) and packed with Obtura S-Kondenser. The canal was backfilled using SuperEndo Beta 2 (B&L Biotech, Ansan, Korea) at a temperature setting of 200 °C. All of the samples were stored in a humidified chamber (Changshin Science, Seoul, Korea) at 100% relative humidity and 37 °C for 14 days until investigated using micro-CT. For standardization, all of the procedures were performed by a single operator.

### 4.3. Micro-CT Imaging and Analysis

After canal preparation, three artificial tooth specimens were randomly selected. Micro-CT images were obtained and overlapped to confirm that the prepared canal space in the artificial teeth was consistent (Figure 1B). A high-resolution micro-CT scanner (SkyScan 1173, Bruker, Billerica, MA, USA) was used to scan the samples. The micro-CT scanner had a pixel size of 11.01 μm; X-ray source voltage, 130 kV; beam current, 60 μA; aluminum filter thickness, 1.0 mm; rotation step, 0.3° per step; and exposure time, 500 ms. Images obtained from the scan were reconstructed using NRecon software version 1.6.6.0 (Bruker microCT, Kontich, Belgium). The range of measurements was 1–5 mm and 5–9 mm from the root apex. The mesial root and distal root were imaged differently, based on the root axis. To evaluate the overall filling state, three-dimensional (3D) images of the filling material were visualized by the surface-rendering program CT-Vol (SkyScan).

The CT-An software (SkyScan) was used to measure the volume of the gap between the filling material and root canal walls, and the voids in the filling material. Three-dimensional image data were obtained after obturation in the *x*, *y*, and *z* axes for the mesial and distal root axes. The most apical 1 mm was not included in the analysis. The area 1–5 mm from the apex constituted the apical area, and the area 5–9 mm from the apex constituted the coronal area. When measuring the voids between the filling material and the root canal wall (V_out_), a gray scale ranging between 40–255 was assigned as the volume of the filling material (V_m_) and a gray scale ranging between 0–40 was assigned as a void. When measuring voids inside the filling material (V_in_), a gray scale ranging between 124–255 was assigned as the volume of the filling material (V_m_), and a gray scale ranging between 0–124 was assigned as a void. The percentage of voids (V_%_) was calculated as follows:

%V_out_ = V_out_/(V_out_ + V_m_) × 100 (1)

%V_in_ = V_in_/(V_in_ + V_m_) × 100 (2)

### 4.4. Statistical Analysis

Shapiro–Wilk and Kolmogorov-Smirnov tests were used to verify whether the data were normally distributed. A linear mixed model (covariance pattern, unconstructed) was used to determine differences between the method effect and the area effect. The two obturation techniques were categorized as follows, based on the area of the voids and the root length: the percentage of the void volume (%V_out_) between the filling material and the root canal wall in the apical 1–5 mm; the percentage of the void volume (%V_in_) inside the filling material in the apical 1–5 mm; the percentage of the void volume (%V_out_) between the filling material and root canal wall in the apical 5–9 mm; and the percentage of the void volume (%V_in_) inside the filling material in the apical 5–9 mm. The significance level was set at *p* < 0.05. Statistical analyses were performed using SPSS version 20 (SPSS Inc., Chicago, IL, USA). 

## 5. Conclusions

The present study demonstrated that the voids between the filling materials and the root canal walls (i.e., %V_out_) were not significantly different between the two obturation techniques in the apical area. Therefore, the SC technique and CW technique were not different with regard to void formation in the apical area.

## Figures and Tables

**Table 1:** The percentage of voids between the filling material and the root canal wall (V_out_).

Canal	Area	SC	CW	Overall *p*-Value
Mesial	Apical	3.86 ± 0.11	3.61 ± 0.11	Method: 0.56 Location < 0.05 * Method × Location < 0.05 *
	Coronal	5.30 ± 0.14	5.48 ± 0.14	
Distal	Apical	3.87 ± 0.12	3.74 ± 0.12	
	Coronal	5.17 ± 0.17	5.72 ± 0.17	

* Indicates a significant difference (*p* < 0.05). The data are presented as the estimated mean ± the standard deviation (*n* = 10 for each group). CW, continuous wave; SC, single cone.

**Table 2:** The percentage of voids inside the filling material (V_in_).

Canal	Area	SC	CW	Overall *p*-Value
Mesial	Apical	4.18 ± 0.36	5.39 ± 0.36	Method: < 0.05 * Location < 0.05 * Method × Location < 0.05 *
	Coronal	2.94 ± 0.77	5.38 ± 0.77	
Distal	Apical	4.00 ± 0.38	5.08 ± 0.38	
	Coronal	3.62 ± 0.76	10.13 ± 0.89	

* Indicates a significant difference (*p* < 0.05). The data are presented as the estimated mean ± the standard deviation (*n* = 10 for each group). CW, continuous wave; SC, single cone.

**Table 3:** Post hoc *p*-values of the methods.

**%V_out_**	Mesial	Apical	0.13
		Coronal	0.40
	Distal	Apical	0.47
		Coronal	<0.05 *
**%V_in_**	Mesial	Apical	<0.05 *
		Coronal	<0.05 *
	Distal	Apical	0.06
		Coronal	<0.05 *
**%V_total_**	Mesial	Apical	0.07
		Coronal	<0.05 *
	Distal	Apical	<0.05 *
		Coronal	<0.05 *

* Indicates a significant difference (*p* < 0.05). %V_in_, percentage of voids inside the filling material; %V_out_, percentage of voids between the filling material and the root canal wall.
